# Hemophagocytic Lymphohistiocytosis Secondary to Epstein-Barr Virus Infection in an Adolescent Male Patient: A Case Report

**DOI:** 10.7759/cureus.95534

**Published:** 2025-10-27

**Authors:** Mehrab Mohaimen Tahseen, Elaina Pasangha

**Affiliations:** 1 Medicine, University Hospitals Bristol and Weston NHS Foundation Trust, Weston-super-Mare, GBR; 2 Hematology, King's College Hospital NHS Foundation Trust, London, GBR

**Keywords:** acute care medicine, bone marrow morphology, epstein-barr virus, hematology, hemophagocytic lymphohistiocytosis

## Abstract

Hemophagocytic lymphohistiocytosis (HLH) is a severe hematological condition characterized by a wide spectrum of clinical presentations, making diagnosis particularly challenging. Potential triggers include infections, malignancies, autoimmune diseases, and certain medications or interventions. We report a case of HLH in an adolescent male in his late teens triggered by Epstein-Barr virus infection. The initial symptoms were malaise, jaundice, and bilateral hearing loss. On examination, the patient had palpable cervical lymphadenopathy, splenomegaly, and epigastric tenderness. Laboratory investigations showed pancytopenia with elevated ferritin and triglyceride levels. The patient was transferred from a district general hospital to the nearest tertiary center and subsequently admitted to the intensive care unit. Bone marrow and lymph node biopsies were performed to exclude lymphoma. He was successfully treated with methylprednisolone, anakinra (interleukin-1 receptor antagonist), and intravenous immunoglobulin, resulting in marked improvement in symptoms, viral load, and biochemical parameters.

## Introduction

Hemophagocytic lymphohistiocytosis (HLH) is a life-threatening condition that activates the immune system, leading to severe inflammation and multi-organ involvement. Given the extensive multi-system involvement of HLH, multidisciplinary teams (MDTs) have been established in London and northern England. These teams include specialists in rheumatology, hematology, neurology, critical care, infectious diseases, immunology, virology, and acute/general medicine [1]. Despite these efforts, access to specialist services for HLH across the United Kingdom remains inconsistent. Data from 2023 highlight a lack of funding, inadequate administrative support, and unsustainable reliance on a single center [2]. Recent data from the United Kingdom indicate a mortality rate of approximately 50%, with a national survival rate of 50% for cases diagnosed between 2003 and 2008 [1]. Data also suggest an increase in the incidence of HLH in England, reflecting improved recognition and diagnosis by physicians as well as a rise in hematological malignancies, inflammatory rheumatic disease, and inflammatory bowel disease [3].

The primary presentations of HLH include patients with a known diagnosis of sepsis or autoimmune disease who subsequently develop HLH-related complications, as well as cases where the condition presents as pyrexia of unknown origin or as an illness characterized by cytopenias. In some cases, the disease may manifest acutely with rapid progression to multi-organ failure, while in others it presents more subacutely with prolonged fever, cytopenias, and evolving systemic involvement [1]. Conditions that can mimic HLH include sepsis, lymphoma, and atypical infections such as visceral leishmaniasis, mycobacterial infections, disseminated adenovirus, and herpes simplex virus, all of which can lead to cytopenias and elevated inflammatory markers [4].

No single test can establish a diagnosis of HLH. Diagnosis requires a combination of clinical features, biochemical parameters, and bone marrow findings, often utilizing the HLH-2004 criteria. These include fever, splenomegaly, cytopenias, hypertriglyceridemia and/or hypofibrinogenemia, hemophagocytosis, low/absent natural killer (NK) cell activity, ferritin >500 μg/L, and elevated soluble interleukin-2 (IL-2) receptor. A score of 5 or more criteria is strongly suggestive of HLH [5].

Management involves rapid control of the hyperinflammatory state alongside treatment of the underlying trigger. Immunosuppression can be achieved using etoposide, dexamethasone, cyclosporine A, and, in select patients, methotrexate and corticosteroids. Allogeneic hematopoietic stem cell transplantation may be considered for primary HLH once the initial hyperinflammatory state is controlled. Relapsed or refractory cases may require cytokine-targeted therapy and immunotherapy, including Janus kinase 1/2 (JAK1/2) inhibitors, anti-CD52 antibodies, anti-CD20 antibodies, and PD-1 blocking agents [6]. Epstein-Barr virus (EBV)-associated HLH can often be managed more conservatively with a course of steroids with or without intravenous immunoglobulin. As EBV replicates in B cells, rituximab may be effective in clearing the viral reservoir. In cases of rapid clinical deterioration, etoposide treatment may be required [7]. Recently, anti-cytokine therapies such as anakinra, an IL-1 antagonist, have been successfully used to treat secondary HLH triggered by rheumatological disease, avoiding the toxic immunosuppressive and myelosuppressive side effects of etoposide [8].

## Case presentation

An adolescent male patient in his late teens presented with jaundice, bilateral hearing loss, and general malaise for three weeks. He had previously been evaluated by a general practitioner and prescribed two courses of antibiotics: amoxicillin and co-amoxiclav. Laboratory testing revealed a positive glandular fever diagnosis and abnormal liver function prior to his presentation at the emergency department. On examination, bilateral palpable submandibular lymph nodes and mild epigastric tenderness were noted. The patient was admitted under the acute medical team with a presumed diagnosis of EBV-induced hepatitis.

**Figure 1:**
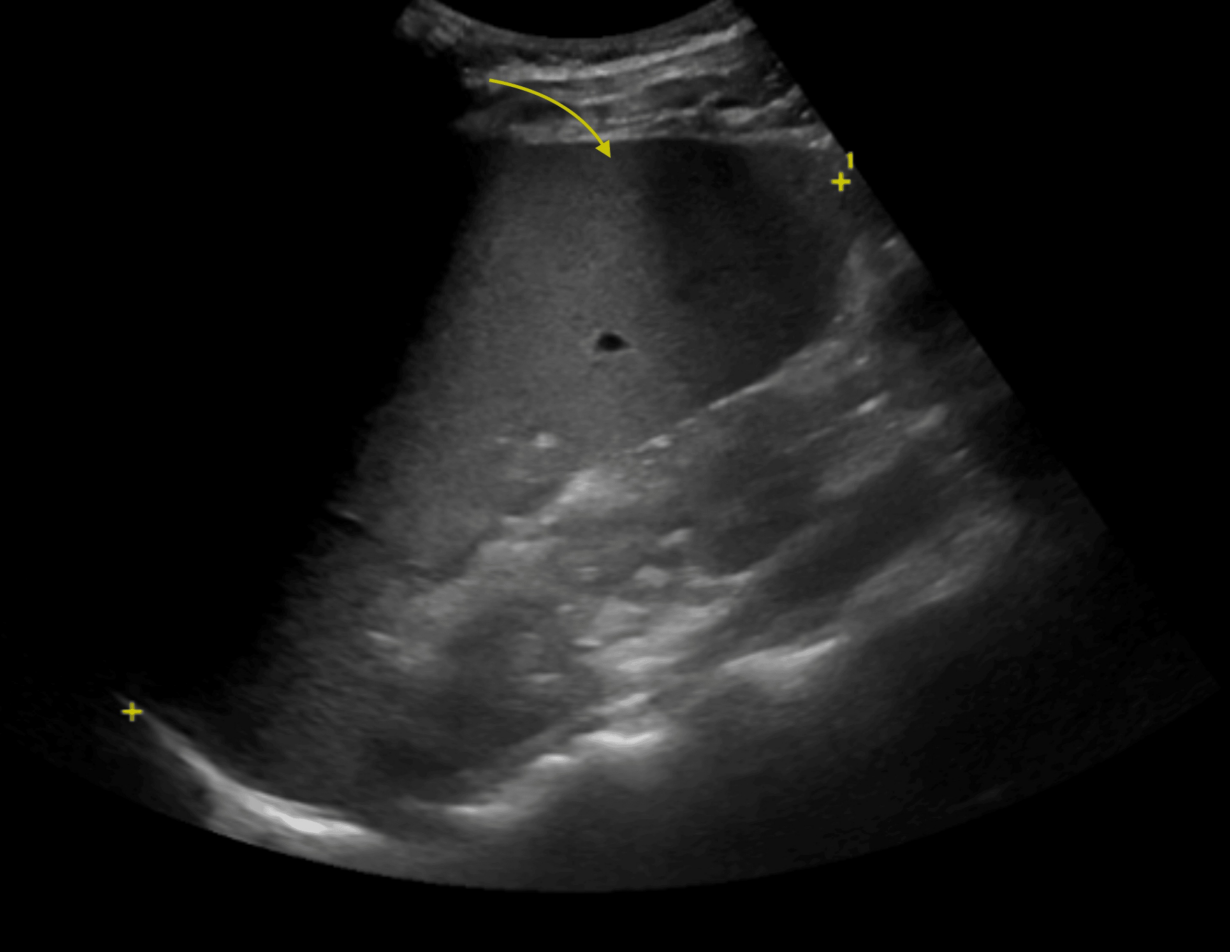
Abdominal ultrasound illustrating splenomegaly

As liver function tests worsened, an abdominal ultrasound revealed splenomegaly measuring 18.6 cm (Figure 1). No obstruction or hepatomegaly was identified. Over the next 24 hours, the patient deteriorated with rapidly rising fever and worsening liver function. These findings prompted consultation with the infectious disease, hematology, and rheumatology teams for suspected HLH. Additional investigations, including triglycerides and ferritin, were ordered. The ear, nose, and throat (ENT) team was consulted for persistent bilateral hearing loss, which they attributed to chronic sinusitis and Eustachian tube dysfunction.

**Figure 2:**
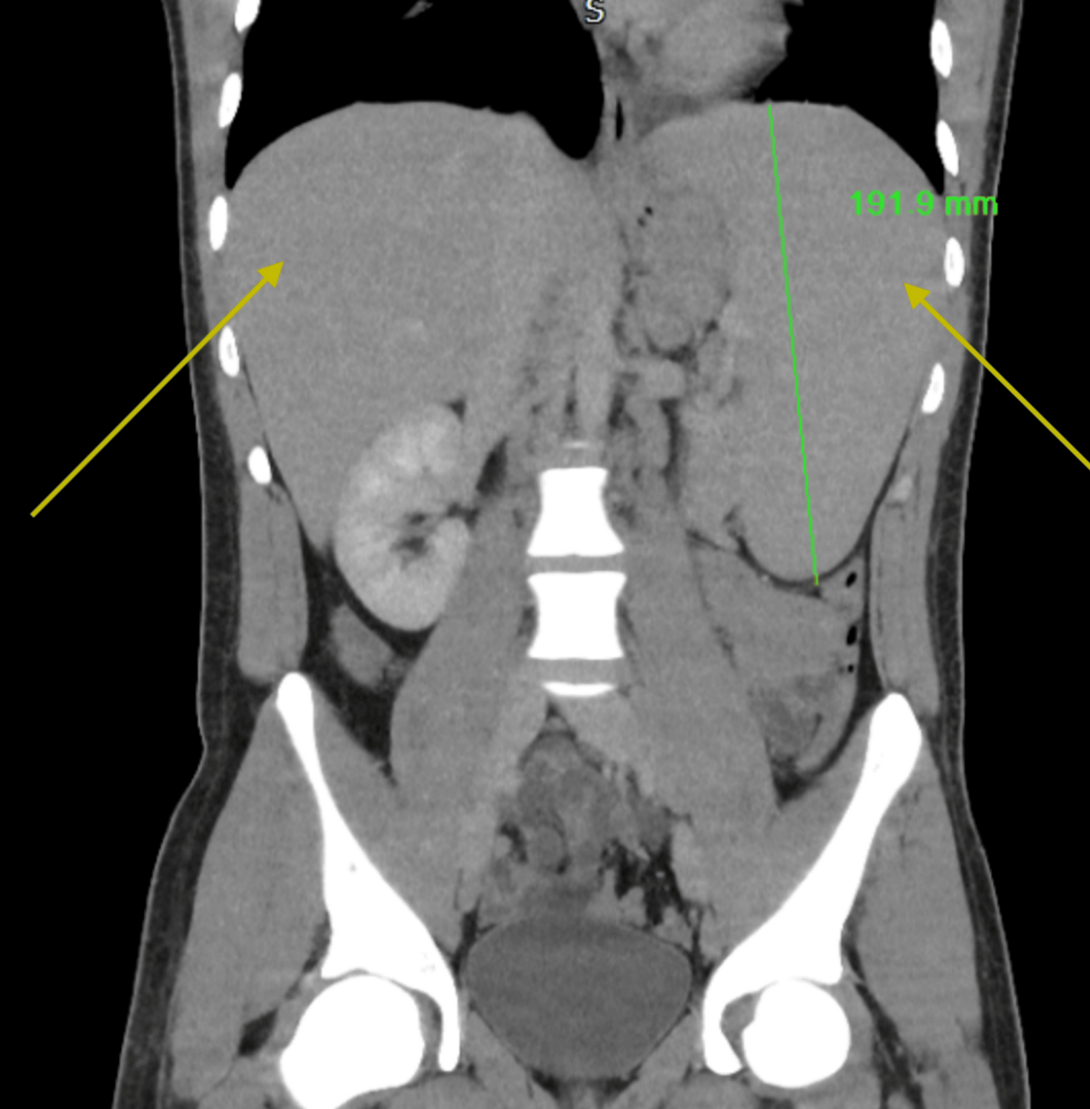
CT of the abdomen demonstrating hepatosplenomegaly CT: computed tomography

**Figure 3:**
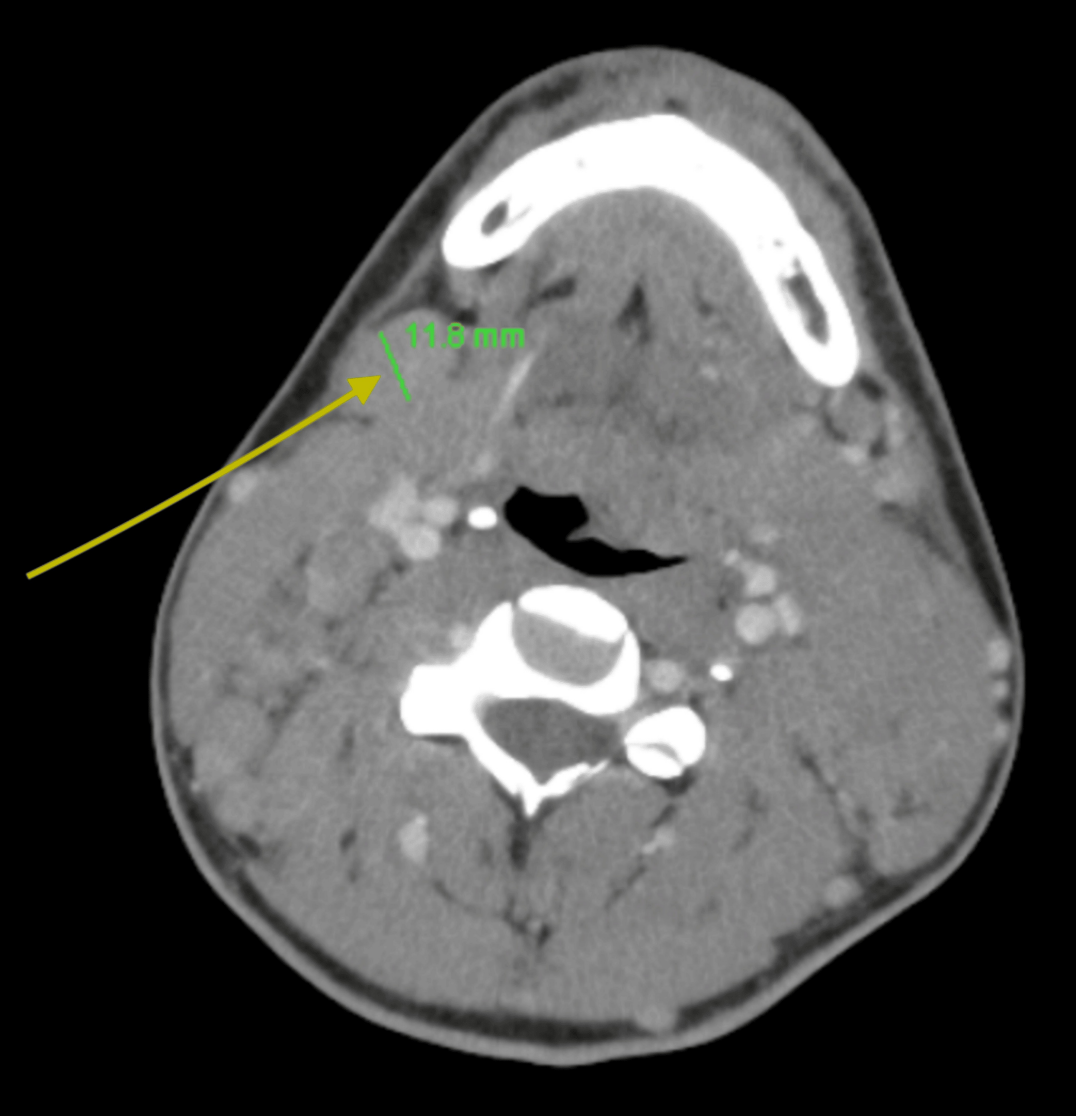
CT of the neck demonstrating enlarged cervical lymph nodes CT: computed tomography

**Figure 4:**
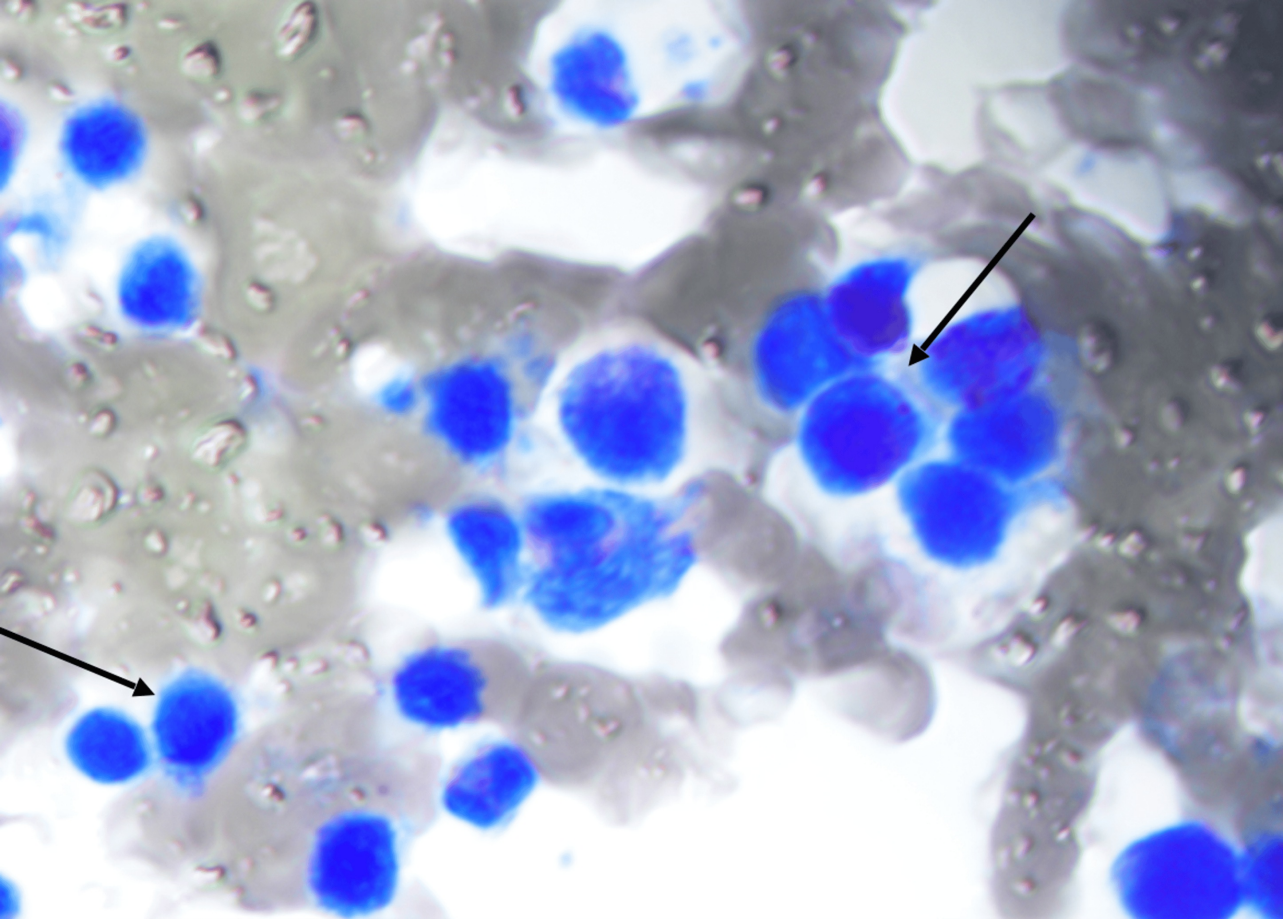
Bone marrow aspirate Bone marrow aspirate showing signs of subtle hemophagocytosis (marked in arrows)

Due to palpable cervical lymph nodes, an urgent computed tomography (CT) scan of the neck, thorax, abdomen, and pelvis was performed to evaluate for lymphoma, as EBV-driven lymphoma was suspected initially. Imaging demonstrated significant hepatosplenomegaly with widespread borderline and enlarged lymphadenopathy in the neck, mediastinum, left axilla, and para-aortic regions (Figure 2 and Figure 3). An urgent cervical lymph node biopsy was performed the following day. After discussion with the HLH MDT, intravenous methylprednisolone at a dose of 1 g daily was initiated, along with proton pump inhibitor and rasburicase prophylaxis, as well as anakinra at 300 mg once daily intravenously. A bone marrow biopsy was also performed, which revealed subtle evidence of hemophagocytosis but no lymphoid infiltrate (Figure 4). The patient's H-score was subsequently calculated to be >90% probability score for HLH, scoring for organomegaly, cytopenia, raised ferritin and triglycerides, and hemophagocytosis on bone marrow aspirate.

**Figure 5:**
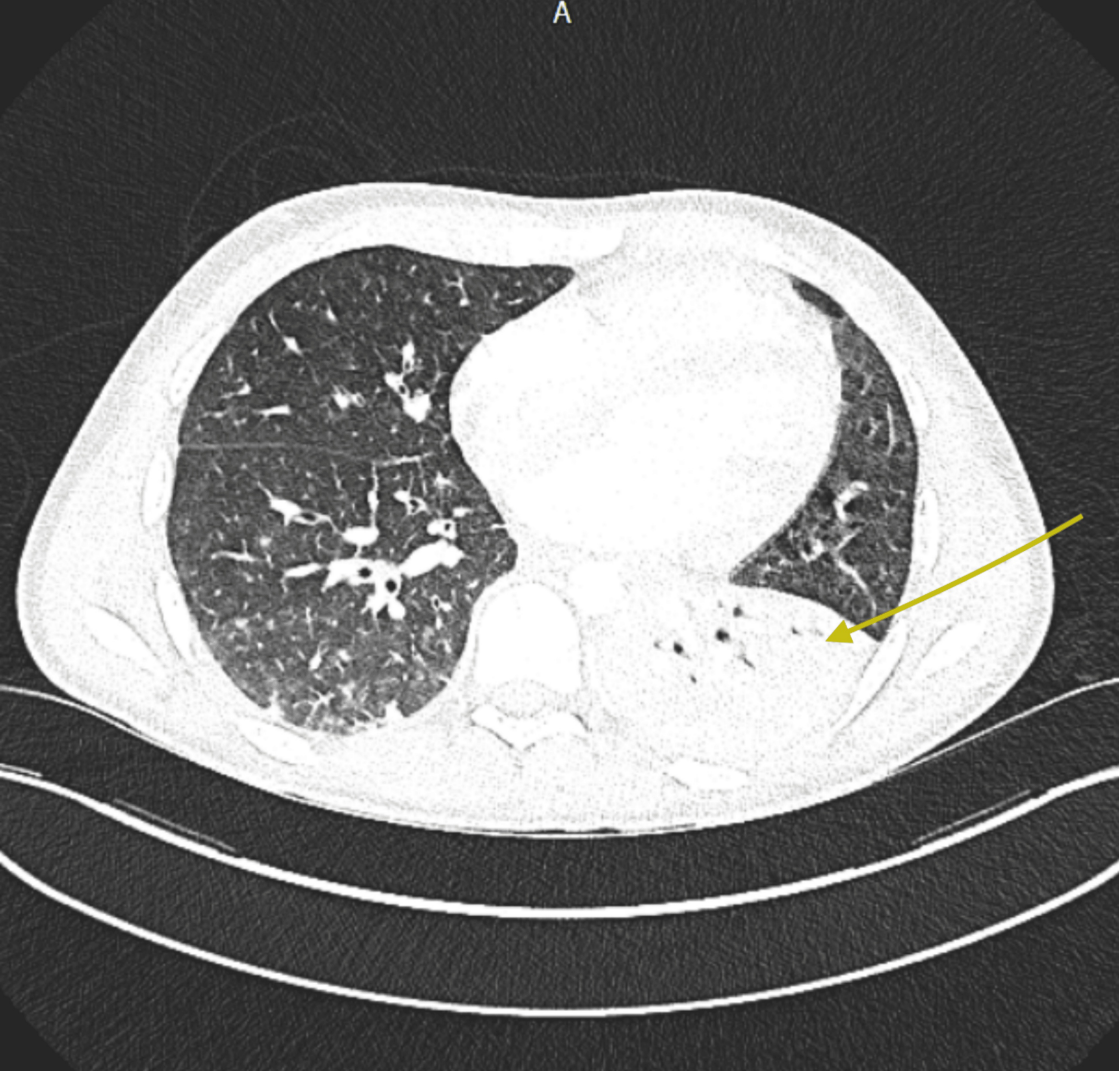
CTPA demonstrating left lobar pneumonia CTPA: computed tomography pulmonary angiogram

Despite treatment, the patient developed worsening oxygen requirements and was admitted to the intensive care unit (ICU) over the next three days. A CT pulmonary angiogram (CTPA) revealed lobar pneumonia and pulmonary embolism (Figure 5). In the context of significant hospital-acquired pneumonia, corticosteroids were withheld, and the patient was continued on a reduced dose of anakinra at 200 mg once daily. Intravenous ceftriaxone was initiated, alongside prophylactic co-trimoxazole and acyclovir. Two doses of intravenous immunoglobulin therapy (1 g/kg) were also administered in the ICU. The cervical biopsy results confirmed infectious mononucleosis with no evidence suggestive of lymphoma. EBV titres were tested regularly and demonstrated a significant reduction in viral load following treatment (Table 1).

**Table 1:** Laboratory data summary CRP: C-reactive protein; EBV: Epstein-Barr virus; ALP: alkaline phosphatase; ALT: alanine aminotransferase; -: not measured

	Day 1	Day 5 (steroids + anakinra commenced)	Day 8 (intravenous immunoglobulin started)	Day 11	Day 55	Units	Reference range
Hemoglobin	110	96	89	86	123	g/dL	130-180
White blood cells	12.2	13.9	6.3	4.7	4.6	10^9^/L	4.0-11.0
Platelets	157	252	200	244	213	10^9^/L	150-450
CRP	59	27	56	13	2	mg/L	<5
Triglycerides	2.9	4.7	4.3	-	4.5	mmol/L	<1.7
Ferritin	5029	1358	1768	1058	269	ng/mL	24-340
EBV viral load	3438260	11756745	327010	201785	<1000	copies/mL	<1000
ALP	440	363	421	411	83	U/L	30-130
ALT	444	184	241	166	65	U/L	<45
Bilirubin	128	26	20	16	7	μmol/L	<21

The patient improved clinically, was transferred back to the ward, and was subsequently discharged home with weekly follow-up in the hematology day unit. He was noted to have frequent upper respiratory tract symptoms prior to hospital admission. Family history of significance included a sibling with Kawasaki vasculitis diagnosed at the age of two years. On discharge, the patient was referred to the regional immunology center for further evaluation and testing to determine the underlying cause of primary immunodeficiency.

## Discussion

The pathogenesis of HLH is attributed to the disruption of the negative feedback loop between NK cells and CD8+ T lymphocytes, which normally induces the apoptosis of antigen-presenting cells (APCs). Consequently, the immune system becomes hyperactive, leading to an excess of pro-inflammatory cytokines, including interferon-gamma (IFN-γ), IL-1, IL-6, and IL-18 [9]. Primary HLH is characterized by the abnormal activation of CD8+ T cells and NK cells, whereas the precise mechanism of secondary HLH remains unclear. The latest Getting It Right First Time (GIRFT) guidelines propose a clinical triad of three "Fs" to aid recognition: persistent fever refractory to antibiotics, falling blood counts (particularly platelets), and elevated ferritin levels. Unfortunately, no evidence-based diagnostic criteria are specifically tailored for secondary HLH [1]. Nevertheless, the H-score can be used to assess the likelihood of HLH, with a reported diagnostic sensitivity of 93% and specificity of 85% [10].

The H-score comprises nine criteria, categorized as follows [5]: clinical criteria (3) (underlying immunosuppression, fever, and organomegaly), biological criteria (5) (ferritin, triglycerides, aspartate aminotransferase, fibrinogen, and cytopenia), and morphological criteria (1) (hemophagocytosis). A total score of 337 can be obtained, with a score exceeding 169 considered diagnostic of HLH. Interpretation of the H-score should be approached with caution in patients with liver failure, as they may have elevated ferritin levels and preexisting cytopenia [5].

The treatment objectives for secondary HLH include suppressing the aberrant activation of the immune system and addressing the underlying trigger. Initial treatment typically involves the administration of corticosteroids with antimicrobial coverage if infection is suspected as the underlying cause. Recommended steroids include intravenous methylprednisolone (1 g/kg) or dexamethasone (10 mg/m² IV), followed by an oral switch to prednisolone (1 g/kg) after 3-5 days [5]. Second-line treatment options include anakinra, a recombinant IL-1 receptor antagonist used off-label for HLH [11]. Dosing typically begins at 1-2 mg/kg/day and can be escalated to a maximum of 8 mg/kg/day. In critically ill patients, intravenous administration may be considered off-label to achieve higher plasma concentrations [12]. Third-line treatment options are determined by MDT discussions and may include intravenous immunoglobulin, cyclosporine, or etoposide [13]. Intravenous immunoglobulin is typically administered at 1 g/kg/day for two days, with the option to repeat the same dose after 14 days if HLH relapses or remains steroid-dependent [1]. Rituximab is often considered in EBV-associated HLH, as EBV tends to replicate in B cells; this approach can improve symptoms, reduce viral load, and dampen inflammation [14].

## Conclusions

HLH is rare and often presents with diagnostic uncertainty. Early recognition, MDT involvement, and tailored immunosuppression are critical for survival. This case illustrates EBV-associated HLH mimicking lymphoma in a young patient. 
